# 

**Published:** 1885-07

**Authors:** 


CONTENTS.

Vol. 32 No. 7

| | PAGE. |
|---|---|
| A Presentiment | 1 |
| How to Lend Money, if you Lend at all | 2 |
| Old London Bridge | 4 |
| The Olive and its Oil | 5 |
| Oil in Storms at Sea | 6 |
| Some Famous Old Maids | 8 |
| The London Ragamuffin | 8 |
| Jimmies | 9 |
| How to Tell a Horse's Age | 9 |
| Cremation in its Legal Aspects | 11 |
| Intestinal Obstruction From Charcoal | 13 |
| The Passion Flower | 13 |
| Tobacco as an Antizymasic | 14 |
| Leprosy | 14 |
| Skunks as Pets | 14 |
| Fear as a Cause of Death | 16 |
| Putting Coins in the Mouth | 16 |
| Treatment of Nervous Dyspepsia by Faradism | 17 |
| Virchow on Cholera | 17 |
| Medical Fantasies | 18 |
| Caulocorea | 20 |

